# Supplementary material for: Evaluation of AlphaFold-Multimer prediction on multi-chain protein complexes
Source: Bioinformatics. 2023 Jul 5;39(7):btad424. doi: 10.1093/bioinformatics/btad424 (PMC10348836; doi:10.1093/bioinformatics/btad424)
Supplement: btad424_Supplementary_Data [file btad424_supplementary_data.pdf]

Supplementary Figures

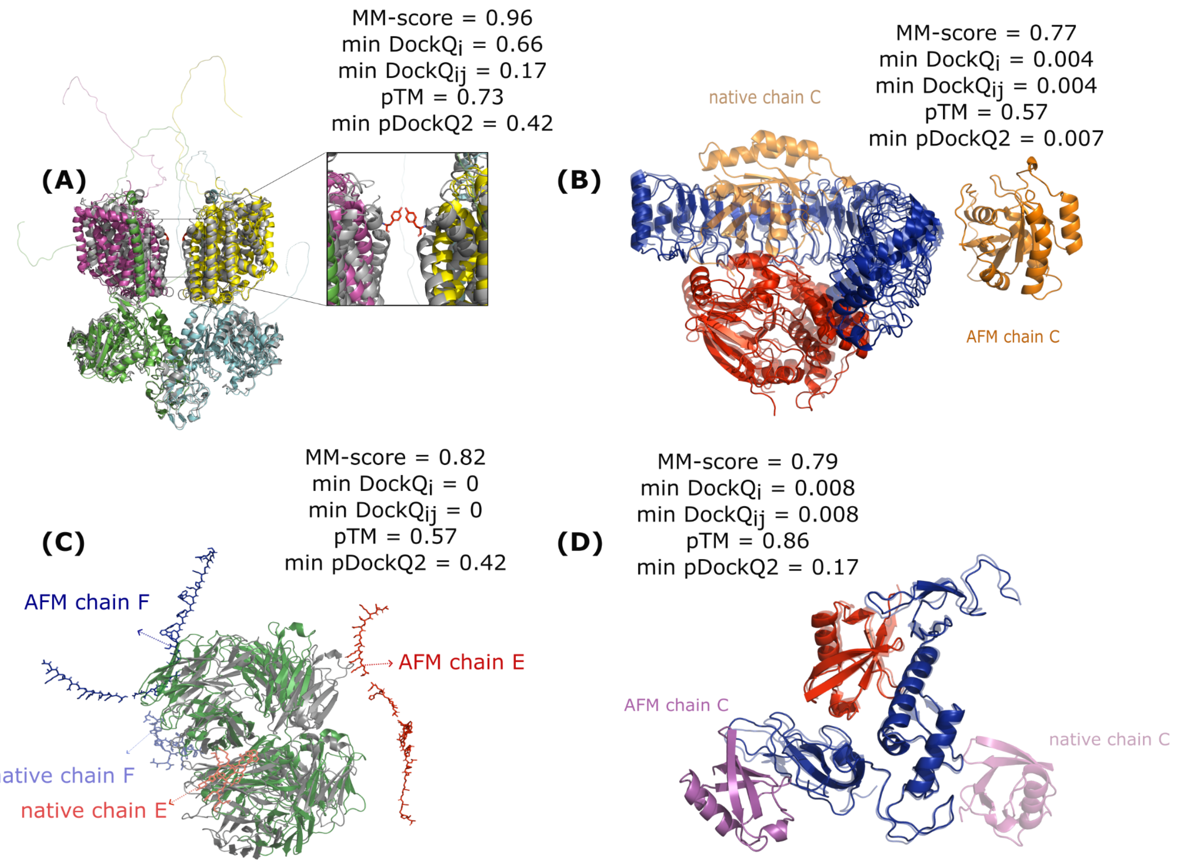

Fig S1. Examples of high variation in different evaluation scores. (a) PDB ID 6LI9 (tetramer) - shows the superimposition of modelled and native structure heteromeric amino acid transporter b0,+AT-rBAT complex bound with arginine (PDB ID: 6LI9). All four $DockQ_i$ give scores above 0.66. A single native contact (shown as sticks in the zoom-in window) between chains C and D was not predicted in the AlphaFold-Multimer model. Hence, the $DockQ_{CD}$ gives 0.17 for this interface despite the two chains looking perfectly aligned with the native structure. (b) PDB ID 7TXH (trimer) - shows the prediction of human MRas Q71R in complex with human Shoc2 LRR domain M173I and human PP1C (the native structure is blurred). The individual chains of this trimer are accurately predicted, and AlphaFold-Multimer only failed to put chain C in the correct position. For this predicted model, $DockQ_i$ from all three interfaces is below 0.11. However, since the interface between chains A and B is correct, the $DockQ_{AB}$ for this interface is 0.43. (c) 6CXG(hexamer). Chain A&B&C&D are in forest green in the AlphaFold-Multimer model, and the native structure is in grey. AlphaFold-Multimer puts chain E and chain F(glycopeptides) far away from the other four chains, so $DockQ_i$ involving these two chains are all 0. (d) 7US1 (trimer) Two out of three chains are correct, and the $DockQ_i$ for the prediction have minimum value 0.08 and maximum value 0.609. The MMscore is 0.790.

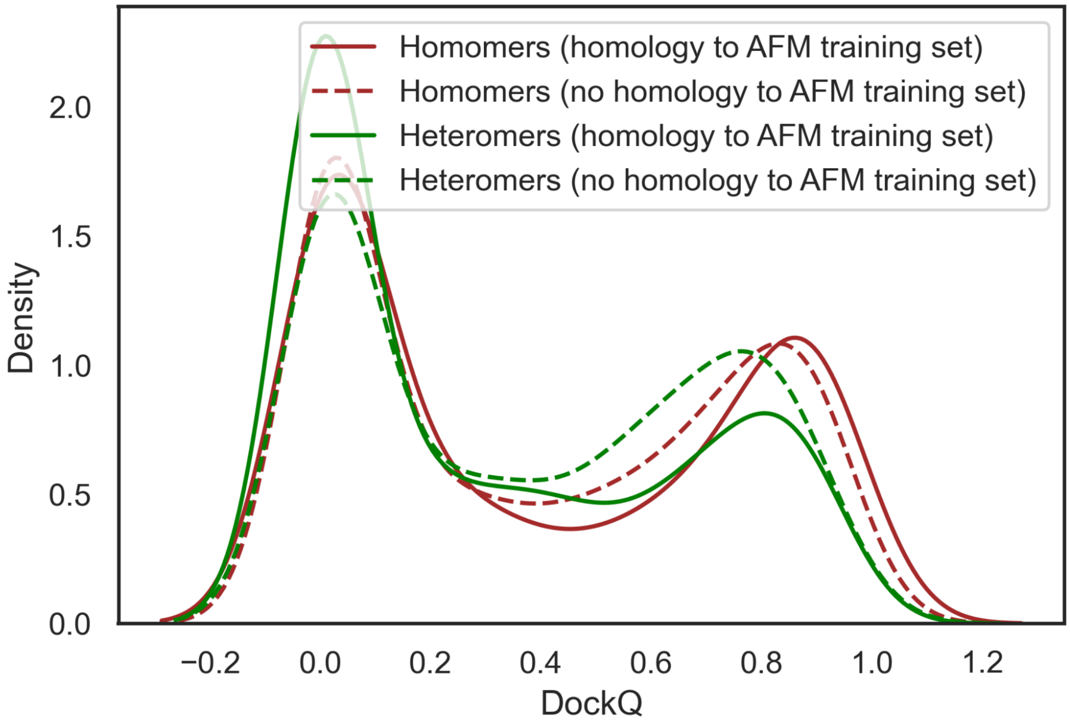

Fig S2. Comparison of DockQ distributions among homomeric and heteromeric complexes on the initial stage of our benchmark dataset, where the only difference is with or without the homology reduction between the AlphaFold-Multimer dataset. The homology reduction procedure is conducted by running HHblits with E-value 10-3 against the UniClust30. If all the chains of a complex are homologous with an older complex (released before 2018-04-30), it is then removed from our dataset.

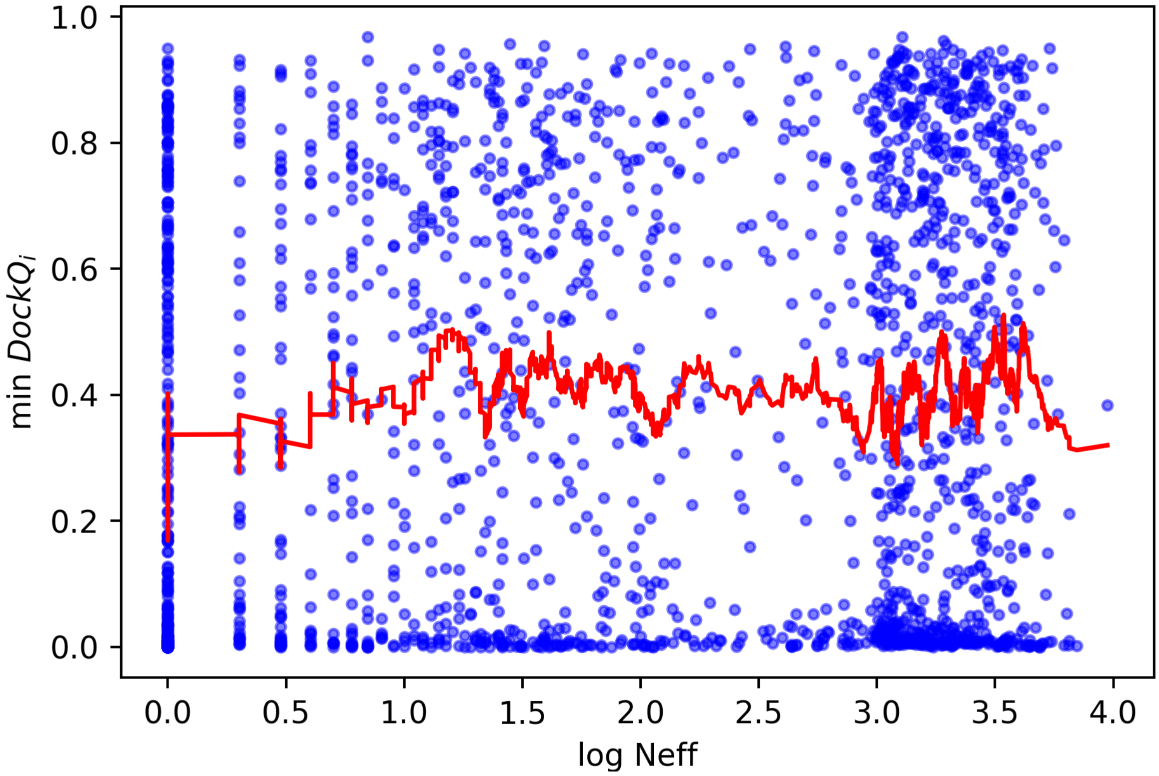

Fig S3. Scatterplot of min $DockQ_i$ and the logarithm of the number of effective sequences (Neff) of paired multiple sequence alignment for each protein complex. The red line shows the running average of min $DockQ_i$.

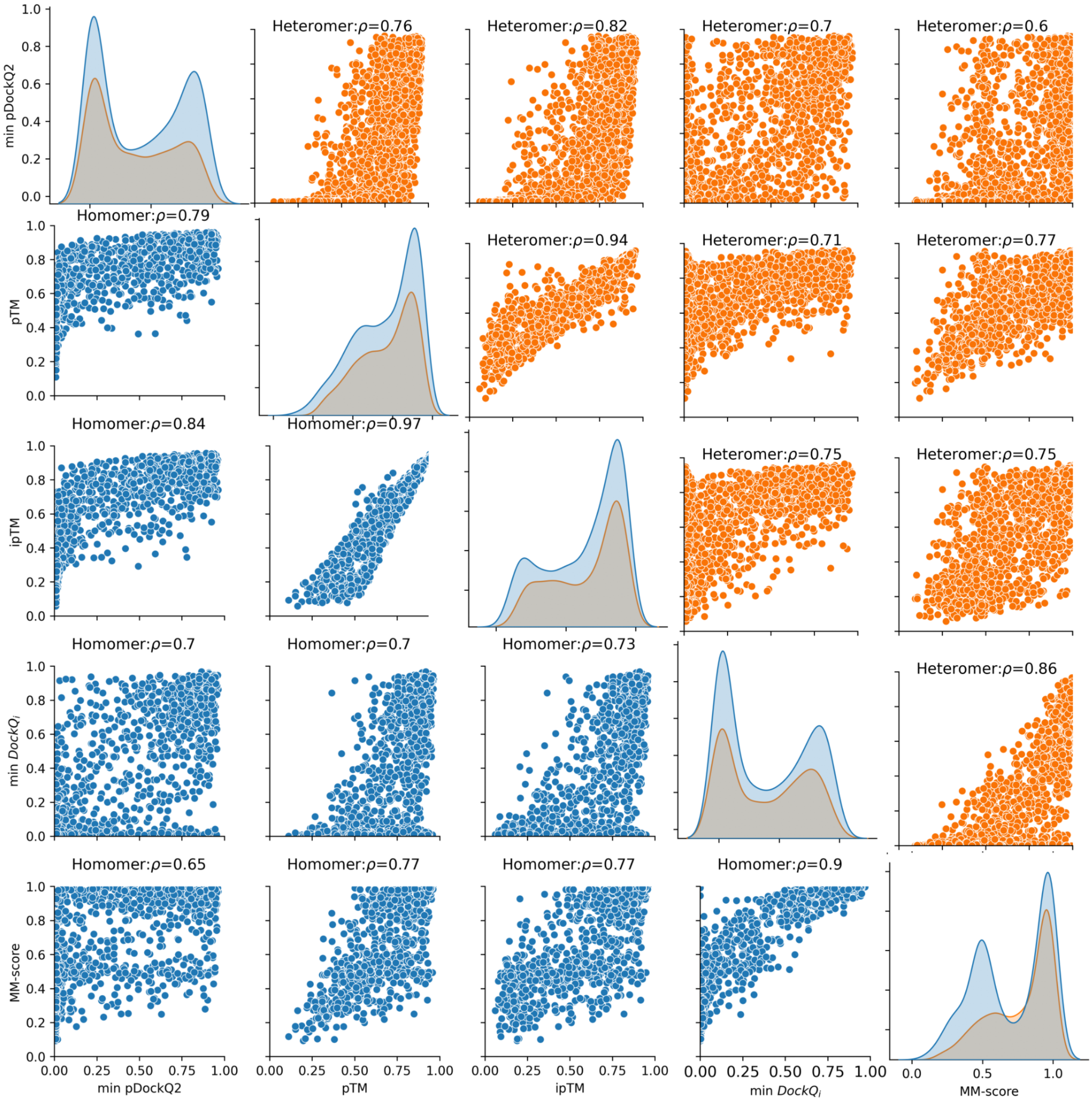

Fig S4. Pairplot showing the correlation between pDockQ2, quality measurements (min $DockQ_i$, MM-score) and confidence scores (pTM, ipTM).

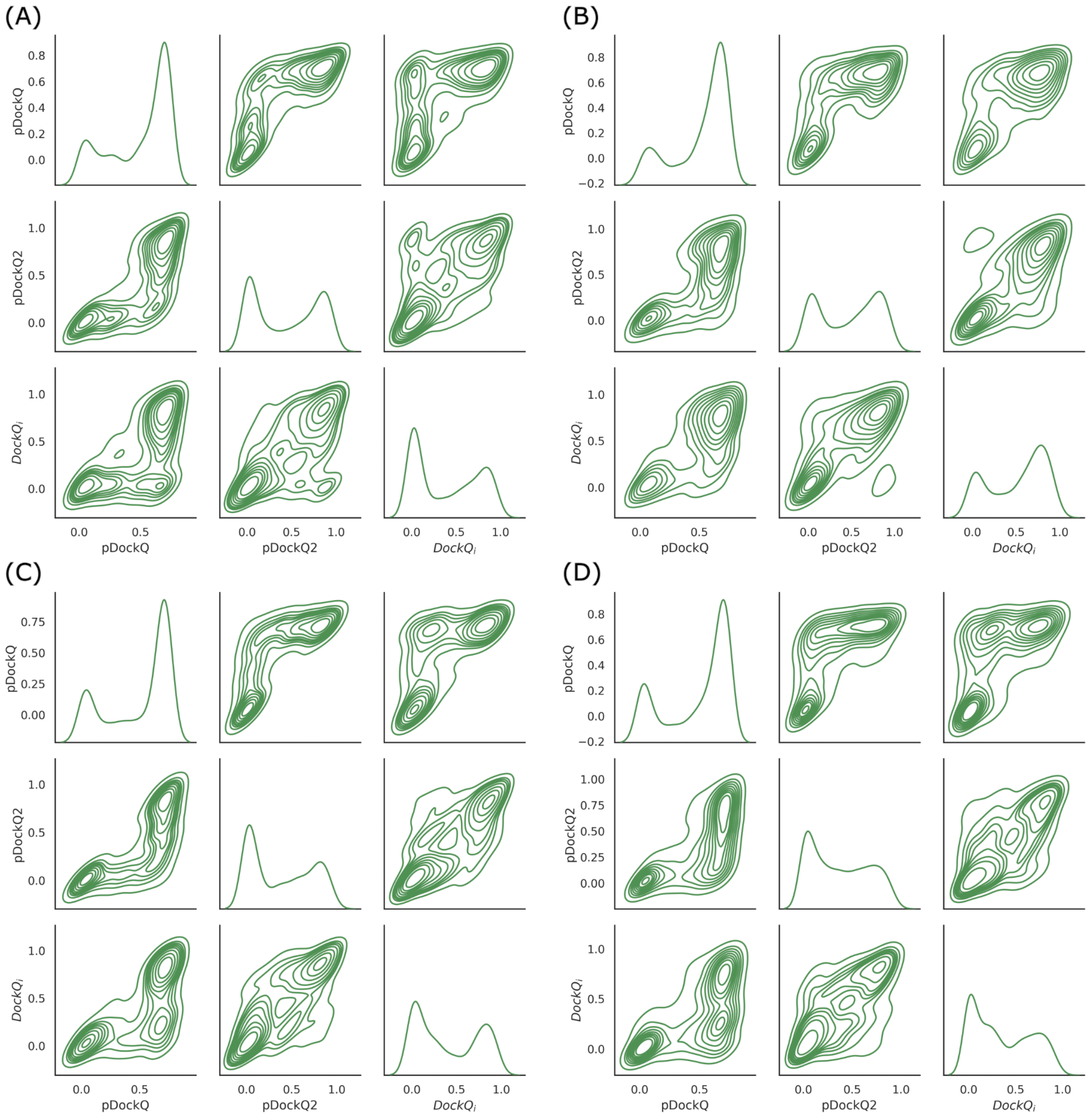

Fig S5. Pairplot representing the relationships between pDockQ, pDockQ2 and DockQ$_i$ for different oligomeric states using AlphaFold-Multimer on the common dataset(n=837). (A) homo-dimers. (B) hetero-dimers. (C) Homomeric protein complexes with three to six chains. (D) Heteromeric protein complexes with the three to six chains.

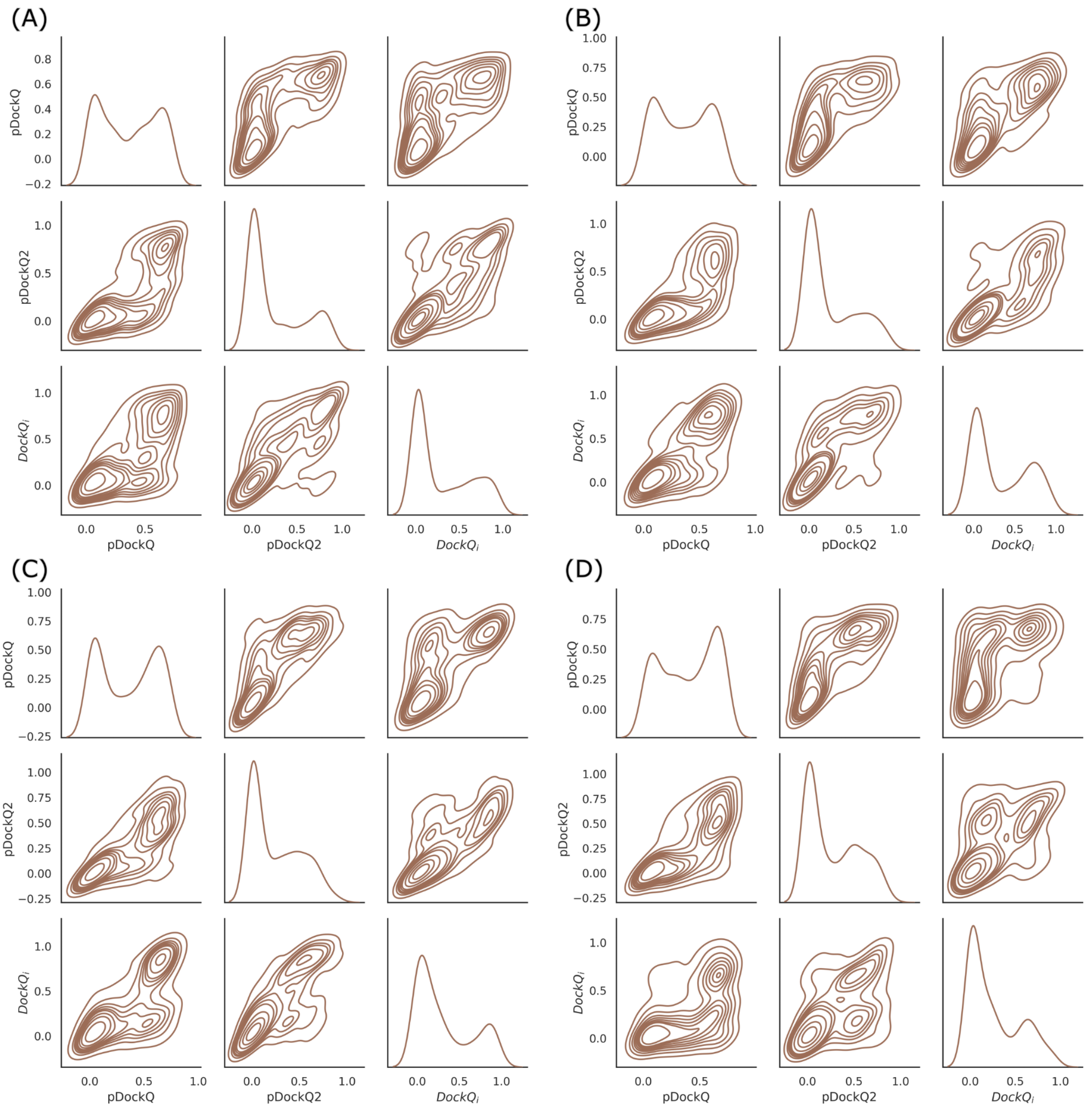

Fig S6. Pairplot representing the relationships between pDockQ, pDockQ2 and $DockQ_i$ for the different oligomeric states using FoldDock on the common dataset (n=837). (A) homo-dimers. (B) hetero-dimers. (C) Homomeric protein complexes with three to six chains. (D) Heteromeric protein complexes with three to six chains.

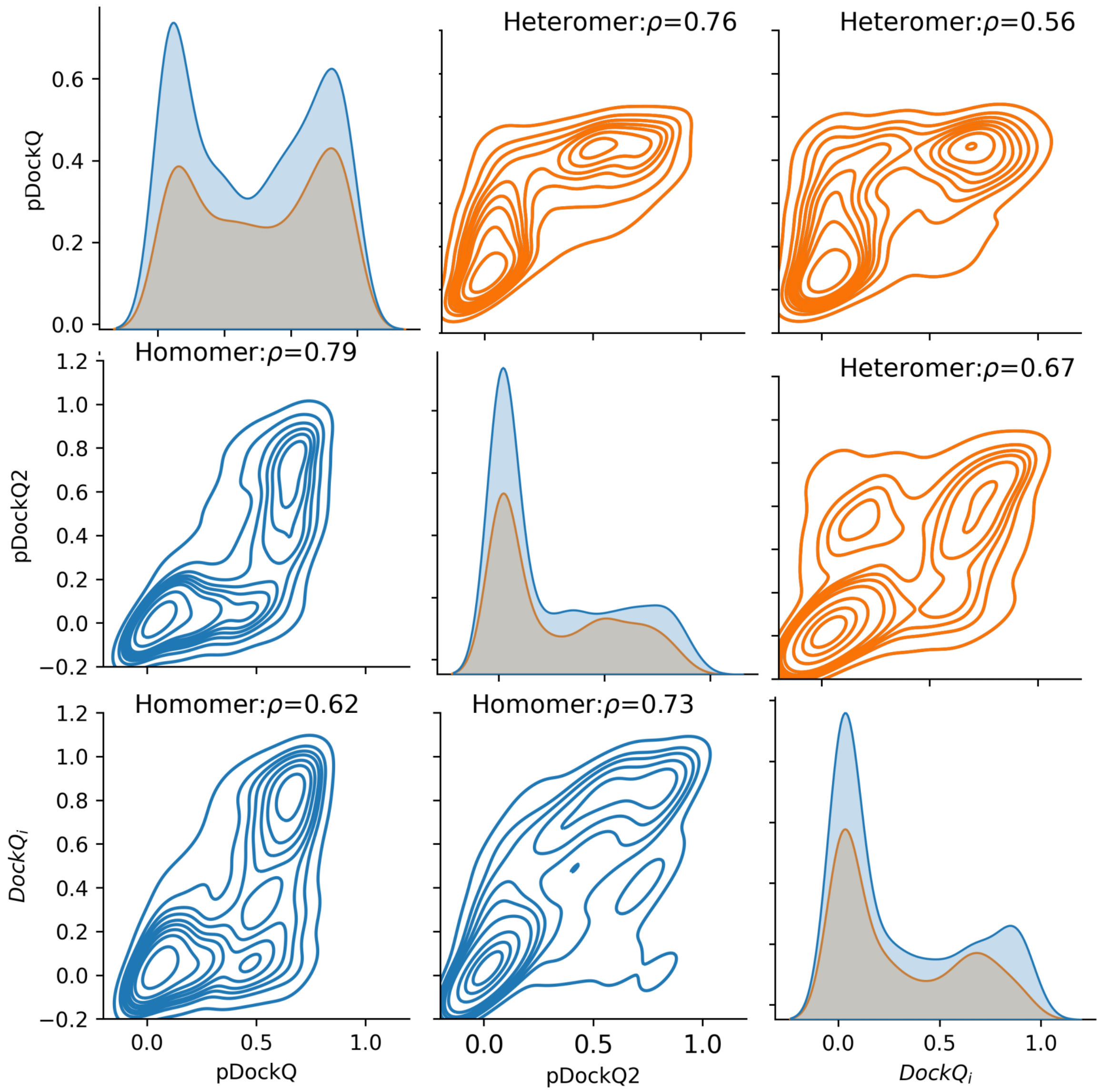

Fig S7. Pairplot showing the relationships between $DockQ_i$ and predicted DockQ scores (pDockQ and pDockQ2) for interfaces of the complexes using FoldDock on the benchmark dataset.

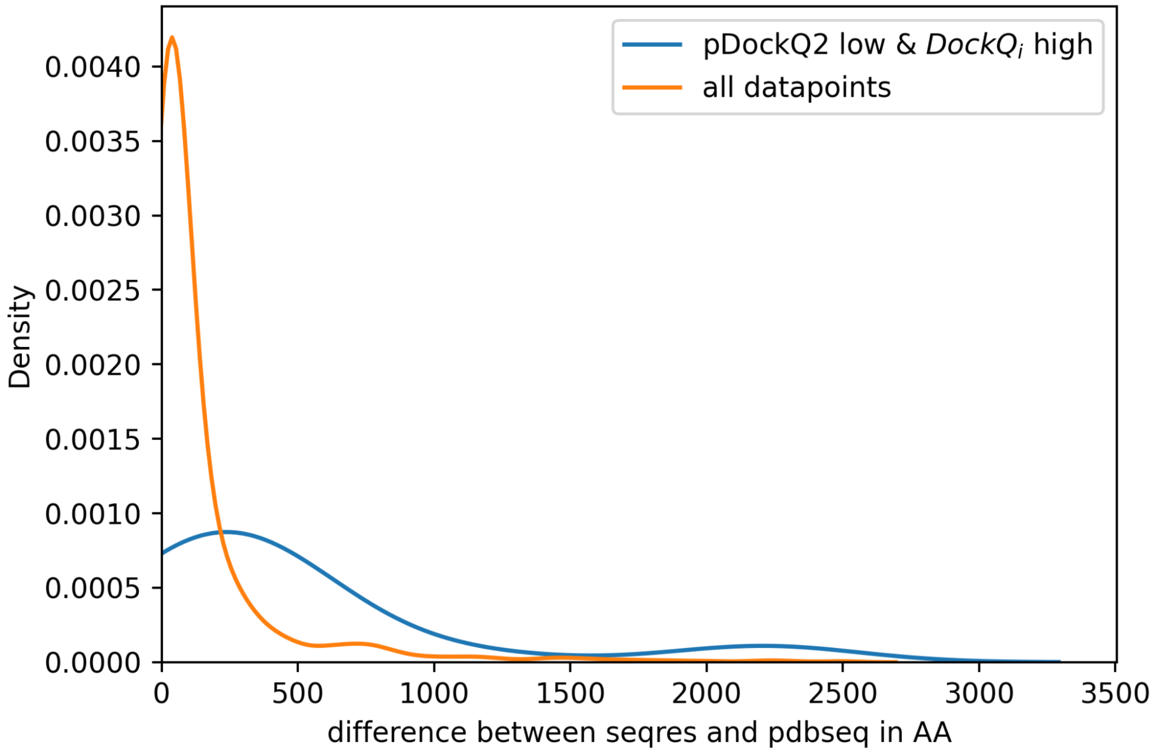

Fig S8: Distribution of difference in SEQRES and PDB chain length for complexes which fit pDockQ2 and those which have low pDockQ2 and high $DockQ_i$

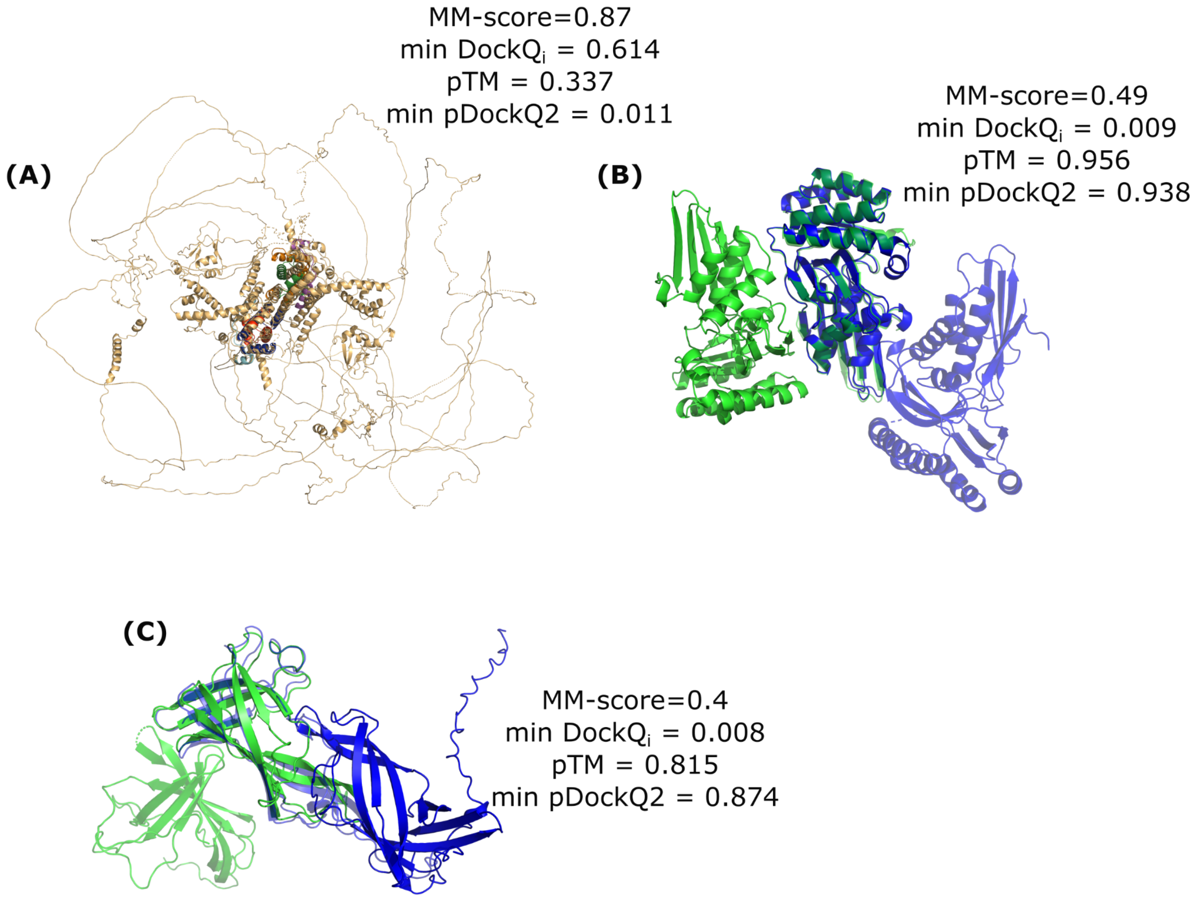

Fig S9: (A) PDB 6XWT, where the native structure is a subset of the model generated from the SEQRES sequence, and the model contains long disordered regions (B) PDB 6JBD - example with high difference in the number of interface contacts in native and model structure (C) PDB 5XLL where chains and native colour the model is coloured in blue and blurry.

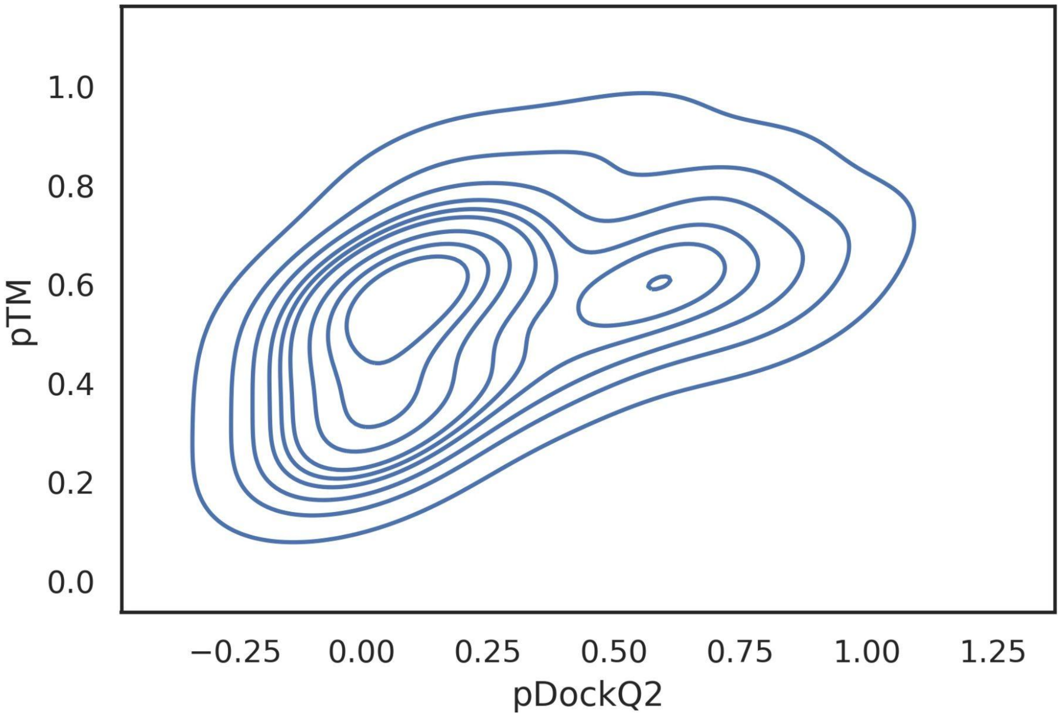

Fig S10. Kernel density plot between pTM (y-axis) and min pDockQ2 scores (x-axis) for the predicted CORUM complexes

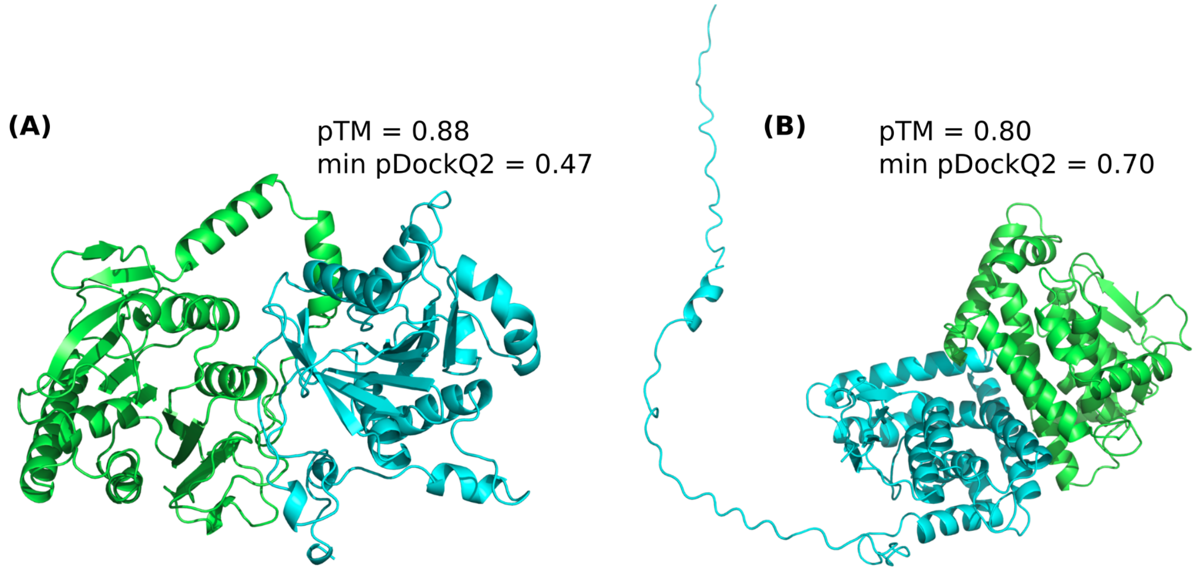

Fig S11. (A) Predicted structure of the PAC1-PAC2 complex (CORUM ID 3034) (B) Predicted structure for the Metaxin complex (Mtx1, Mtx2) (CORUM ID 3094)

Supplementary Tables

Table S1. Success rates (i.e. the fraction of acceptable interfaces with $DockQ_i \geq 0.23$) for protein complex predictions by AlphaFold-Multimer

	2mer	3mer	4mer	5mer	6mer
Homomer	55.5% (n=838)	60.1% (n=86)	60.4% (n=182)	60.0% (n=20)	47.0% (n=55)
Heteromer	72.9% (n=310)	65.9% (n=134)	58.6% (n=185)	42.4% (n=42)	52.4% (n=76)
Overall	60.2% (n=1148)	63.7% (n=220)	59.5% (n=367)	48.1% (n=62)	50.1% (n=131)

Table S2. Success rates (i.e. the fraction of acceptable interfaces with $DockQ_{ij} \geq 0.23$) for protein complex predictions by AlphaFold-Multimer

	2mer	3mer	4mer	5mer	6mer
Homomer	55.5% (n=838)	57.7% (n=86)	52.9% (n=182)	45.7% (n=20)	30.5% (n=55)
Heteromer	72.9% (n=310)	59.3% (n=134)	46.5% (n=185)	34.0% (n=42)	36.3% (n=76)
Overall	60.2% (n=1148)	58.6% (n=220)	49.7% (n=367)	38.2% (n=62)	33.6% (n=131)

Table S3. Success rates (i.e. the fraction of acceptable models with min $DockQ_i \geq 0.23$) for protein complex predictions by AlphaFold-Multimer

	2mer	3mer	4mer	5mer	6mer
Homomer	55.5% (n=838)	59.3% (n=86)	56.0% (n=182)	60.0% (n=20)	45.5% (n=55)
Heteromer	72.9% (n=310)	52.2% (n=134)	45.9% (n=185)	26.2% (n=42)	42.1% (n=76)
Overall	60.2% (n=1148)	55% (n=220)	50.9% (n=367)	37.1% (n=62)	43.5% (n=131)

Table S4. Success rates (i.e. the fraction of acceptable models with max $DockQ_i \geq 0.23$) for protein complex predictions by AlphaFold-Multimer.

	2mer	3mer	4mer	5mer	6mer
Homomer	55.5% (n=838)	60.5% (n=86)	63.7% (n=182)	60.0% (n=20)	50.9% (n=55)
Heteromer	72.9% (n=310)	79.1% (n=134)	74.1% (n=185)	64.3% (n=42)	64.5% (n=76)
Overall	60.2% (n=1148)	71.8% (n=220)	68.9% (n=367)	62.9% (n=62)	58.8% (n=131)

Table S5. Success rates (i.e. the fraction of acceptable models with MMscore > 0.75) for protein complex predictions by AlphaFold-Multimer.

	2mer	3mer	4mer	5mer	6mer
Homomer	50.6% (n=838)	61.6% (n=86)	44.5% (n=182)	55% (n=20)	40% (n=55)
Heteromer	76.8% (n=310)	64.9% (n=134)	48.6% (n=185)	38% (n=42)	43.4% (n=76)
Overall	57.7% (n=1148)	63.6% (n=220)	46.6% (n=367)	43.5% (n=62)	42% (n=131)

Table S6. Success rates (i.e. the fraction of acceptable models with min $DockQ_i \geq 0.23$) for protein complex predictions by AlphaFold-Multimer, FoldDock, Haddock and OmegaFold for the common subset(n=837).

Oligomeric state	AlphaFold-Multimer	FoldDock	OmegaFold	ESMFold
Dimer	60.2%	44.4%	23.9%	28.3%
Trimer	55.0%	33.0%	12.6%	12.7%
Tetramer	51.0%	36.8%	16.7%	12.4%
Pentamer	37.1%	33.3%	16.7%	8.7%
Hexamer	43.5%	30.8%	11.5%	5.1%

Table S7. PDB IDs with high (>0.85) pDockQ2 (i.e. highly confident predictions by AlphaFold) and low (<=0.23) $DockQ_i$ scores. Possibly incorrect biological unit in PDB

PDB ID	Number of chains	Class	MM-score	pTM	ipTM	Symmetry	min $DockQ_i$	min pDockQ2
5xll	2	homomer	0.4	0.815	0.794	C2	0.008	0.874
5yek	2	homomer	0.494	0.827	0.823	C2	0.01	0.909
5ze7	2	homomer	0.489	0.937	0.925	C1	0.006	0.922
6ea8	2	homomer	0.497	0.859	0.847	C2	0.007	0.903
6eg7	2	homomer	0.494	0.899	0.905	C2	0.013	0.887
6gf6	2	homomer	0.581	0.765	0.748	C1	0.026	0.928
6jbd	2	homomer	0.494	0.956	0.947	C2	0.009	0.938
6k62	2	homomer	0.494	0.921	0.907	C2	0.006	0.861
6kew	2	homomer	0.491	0.896	0.876	C1	0.002	0.911
6noy	2	homomer	0.511	0.551	0.552	C2	0.01	0.924
6sm4	2	homomer	0.498	0.763	0.718	C2	0.016	0.919
6t4d	2	homomer	0.507	0.861	0.856	C2	0.006	0.93
6tgp	2	homomer	0.508	0.86	0.84	C1	0.03	0.926
6uxu	2	homomer	0.498	0.936	0.915	C2	0.006	0.911
6vd8	2	homomer	0.499	0.876	0.862	C2	0.024	0.942
7cf7	2	homomer	0.428	0.915	0.897	C2	0.025	0.936
7ebs	2	homomer	0.475	0.929	0.915	C2	0.008	0.864
7f9i	2	homomer	0.523	0.726	0.703	C1	0.042	0.945
7kpo	2	homomer	0.279	0.673	0.712	C2	0.01	0.885
7wsj	2	homomer	0.48	0.784	0.741	C2	0.025	0.903
5z5o	2	heteromer	0.772	0.868	0.858	C1	0.007	0.946
6dxo	2	heteromer	0.526	0.709	0.76	C1	0.223	0.922
6lki	2	heteromer	0.614	0.898	0.878	C1	0.02	0.898
6nep	2	heteromer	0.643	0.894	0.869	C1	0.014	0.87
6oqj	2	heteromer	0.667	0.826	0.845	C1	0.169	0.92
7b2i	2	heteromer	0.507	0.919	0.939	C1	0.006	0.875
7pku	2	heteromer	0.603	0.781	0.851	C1	0.22	0.852
6k6i	3	homomer	0.332	0.926	0.915	C3	0.01	0.9
6yaj	4	homomer	0.594	0.8	0.759	D2	0.188	0.931
7ddy	4	homomer	0.743	0.897	0.891	C1	0.009	0.954
7mq1	6	homomer	0.359	0.813	0.785	D3	0.011	0.877

Table S8. Modelled CORUM IDs having no homology to existing structures

CORUM ID	num_chains	Description	pTM	ipTM	min pDockQ2
12	3	BLOC-2 (biogenesis of lysosome-related organelles complex 2)	0.403	0.357	0.021
118	4	GPI-GnT activity complex	0.563	0.57	0.608
325	4	-	0.467	0.398	0.009
326	3	-	0.573	0.572	0.106
332	4	-	0.502	0.476	0.013
334	4	-	0.67	0.661	0.182
342	5	-	0.622	0.603	0.01
902	3	-	0.4	0.462	0.258
967	2	-	0.351	0.27	0.031
1057	2	DIPA-MCRS1 complex	0.281	0.094	0.009
1064	3	IFP35-NMI complex	0.608	0.527	0.028
1388	2	-	0.505	0.422	0.01
3020	2	-	0.22	0.143	0.008
3034	2	PAC1-PAC2 complex	0.879	0.886	0.47
3094	2	Metaxin complex (Mtx1, Mtx2) complex	0.798	0.86	0.702
3262	3	SCAMP1-SCAMP2-SCAMP3 complex	0.584	0.544	0.031
3525	4	COG subcomplex (COG5, COG6, COG7, COG8)	0.43	0.447	0.433
3532	2	COG1-COG8 subcomplex	0.685	0.705	0.622
3943	2	-	0.322	0.218	0.008
6407	2	-	0.667	0.656	0.115
6415	3	pallidin-Cappuccino-BLOS1 complex	0.585	0.619	0.425
6416	3	dysbindin-Snapi	0.536	0.684	0.834

		n-BLOS2 complex			
6441	3	KIAA0753-FOR20-OFD1 complex	0.281	0.279	0.016
6458	2	TIM23(sort) subcomplex (TIMM17B, TIMM23)	0.683	0.744	0.878
6626	4	AP5 adaptor complex	0.615	0.576	0.189
6695	2	MKKS-BBS12 complex	0.829	0.898	0.108
6847	2	DBIRD complex	0.405	0.152	0.008
7411	5	-	0.609	0.648	0.521
